# Membranes Fouling Propensity of PSF/GO Hollow Fiber Mixed Matrix Membranes for Water Treatment Ultrafiltration Application

**DOI:** 10.1002/cssc.202401061

**Published:** 2024-11-28

**Authors:** Jeanne Casetta, Héloïse Baldo, Laurence Soussan, Céline Pochat‐Bohatier, Mikhael Bechelany, Philippe Miele

**Affiliations:** ^1^ Institut Européen des Membranes, IEM, UMR-5635 Univ Montpellier, ENSCM, CNRS Place Eugene Bataillon 34095 Montpellier France; ^2^ Functional Materials Group Gulf University for Science and Technology (GUST) Mubarak Al-Abdullah 32093 Kuwait

**Keywords:** Membrane, Hollow fibers, Graphene oxide, Polysulfone, Fouling

## Abstract

The study focused on investigating the fouling propensity of polysulfone (PSF) hollow fiber (HF) mixed matrix membranes modified with 1.0 wt % graphene oxide (GO). Through a comprehensive set of analyses including scanning electron microscopy (SEM), atomic force microscopy (AFM), water contact angle (WCA), and mechanical assessments, the structural characteristics and properties of both untreated and GO‐modified PSF HF membranes were thoroughly examined. The scope of evaluation encompassed filtration and separation experiments involving not only pure water but also a range of model contaminants with distinct sizes, such as bovine serum albumin (BSA), humic acid (HA), *E. coli* bacteria, and oil‐in‐water emulsion. Remarkably, the incorporation of graphene oxide (GO) into the PSF HF membranes led to a substantial enhancement in their antifouling performance. The GO‐modified membranes exhibited an impressive recovery rate of over 90 % of their initial pure water flux during filtration experiments involving humic acid (HA) and oil, demonstrating their exceptional resistance to irreversible fouling. Moreover, the GO‐modified membranes exhibited superior oil separation efficiency, further underscoring their efficacy in real‐world separation applications. It is noteworthy that the fouling parameters in the case of bovine serum albumin (BSA) were relatively similar for both the unmodified and GO‐modified PSF HF membranes. This observation suggests that the introduction of graphene oxide might not significantly influence the interaction between the membrane and BSA molecules. Interestingly, while both types of PSF HF membranes displayed high retention capabilities for *E. coli* bacteria, the addition of graphene oxide did not result in any noticeable improvement in this regard.

## Graphical abstract

The study focus on investigating the antifouling performance of polysulfone hollow fiber mixed matrix membranes modified with 1.0 wt % graphene oxide. The modified membranes exhibited an impressive recovery rate of over 90 % of their initial pure water flux during filtration experiments involving humic acid (HA) and oil, demonstrating their exceptional resistance to irreversible fouling.

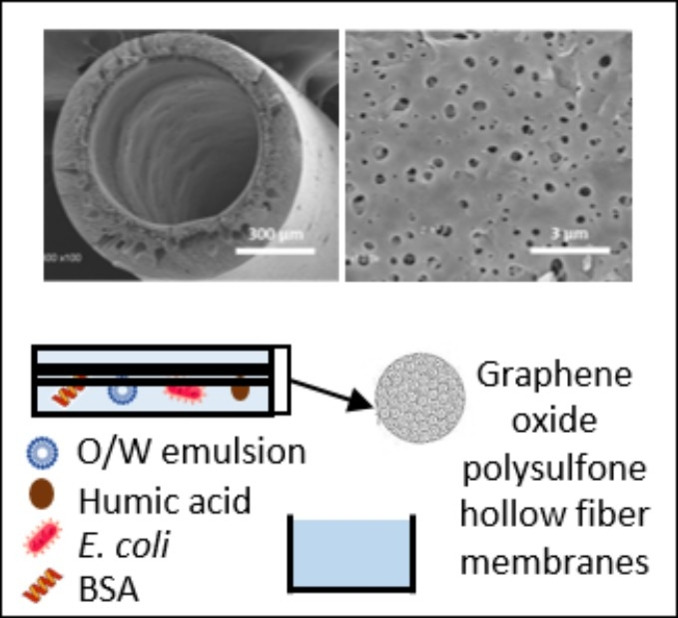

## 1 Introduction

Until now, numerous methods for water purification have been devised to isolate pollutants from water sources. Notably, membranes have emerged as cutting‐edge technologies, owing to their numerous benefits which encompass remarkable separation efficiency, user‐friendliness, minimal energy consumption, and environmentally conscious characteristics. According to the separation characteristics such as water permeability and selectivity, the separation process of pressure operation can be divided into four categories: microfiltration (MF), ultrafiltration (UF), nanofiltration (NF) and reverse osmosis (RO). Membranes pores structure and size of membranes which will influence their filtration performances highly depends on the way they are synthesized. These past few years, hollow fiber (HF) membranes attracted interest due to their larger specific area, excellent scalability and superior flexibility when compared to flat membranes.^[1]^ Numerous synthetic polymers can be used in membrane preparation such as polyethylene (PE), polyethersulfone (PES), polyvinylidene fluoride (PVDF) or polysulfone (PSF). The latter option stands as a prime selection for fabricating HF membranes due to its exceptional chemical and thermal resilience during membrane cleaning, coupled with its commendable mechanical traits that align well with prolonged usage requirements. Nonetheless, a primary drawback associated with this polymer lies in its inherent hydrophobic nature, which notably impacts its antifouling capabilities. Indeed as natural organic matter (NOM), the main foulant present in water, is most of the time hydrophobic, it will tend to adhere easily on hydrophobic surfaces.^[2]^

Several modification techniques suitable for polymeric membranes[^3^, ^4^] have been developed to offset this problematic like grafting or coating for instance. In previous study, we deposited TiO_2_ via atomic layer deposition (ALD) on PSF HF membranes and observed 50 % higher water permeability and 20 % enhanced fouling resistance against bovine serum albumin (BSA) of the PSF HF membranes with only 20 ALD cycles. Concurrently, this was accompanied by an increase augmentation in hydrophilicity and a decrease in pore size.^[5]^ Utilizing mixed matrix membranes (MMMs) presents another auspicious approach to addressing hydrophobicity concerns. This involves the incorporation of hydrophilic fillers into the polymer matrix, thereby enhancing various membrane attributes such as hydrophilicity, strength, permeability, and antifouling characteristics. Zhang *et al*. developed a synergistic antibacterial mixed matrix membrane by integrating guanidyl‐functionalized graphene nanosheets into a PSF matrix through a phase inversion technique.^[6]^ This resulted in guanidyl‐functionalized graphene/PSF mixed matrix membranes (GFG/PSF MMMs) that outperformed pure PSF membranes. The GFG/PSF MMMs exhibited superior permeability and notable antifouling properties against BSA. Moreover, these membranes demonstrated remarkable antimicrobial activity against *E. coli* and *S. aureus*, while maintaining their efficacy over an extended duration.

Among the most widely reported nanoparticles, including TiO_2_, ZnO or SiO_2_, graphene oxide (GO) has received increasing attention because of its exceptionally high crystal, thermal, mechanical and electronic quality.^[7]^ In fact, GO is an atomic‐layer thick 2D material that has lately attracted interest[^8^, ^9^] as it displays a noteworthy hydrophilicity due to its oxygen‐rich functional groups that deserves to be explored for water treatment application.[^10^, ^11^] These properties make GO nanosheets an outstanding candidate for designing antibacterial membranes.^[12]^ Their oxygenated groups, able to generate oxidative stress, exhibited promising antibacterial properties associated with the contact of the bacterial outer membrane.^[13]^ Actually, graphene‐based membranes have been already studied and first results are encouraging because they showed that GO addition lead to notably improved performances in terms of permeability, mechanical and antifouling properties.[^14^, ^15^, ^16^, ^17^, ^18^] In fact, Ionita *et al*. prepared PSF‐GO MMMs by classical phase inversion method and focused on their thermal and mechanical properties. The Raman spectroscopy, X‐ray diffraction and TEM investigations indicated an excellent dispersability with PSF matrix for the low content GO (0.25, 0.5 and 1 wt %). The MMMs present a higher thermal stability and enhanced mechanical properties with the lowest (0.25 and 0.5 wt %) amount of GO with respect to neat PSF. This confirmed the formation of a strong interface necessary for an efficient load transfer from the PSF matrix to the GO.^[19]^ Park *et al*. synthesized thin film composite (TFC) forward osmosis (FO) membranes with GO‐modified support layer. Results revealed that 0.25 wt % of GO addition is the optimal amount with favorable structural property measured in terms of thickness, porosity and pore size. The optimum incorporation of GO in the PSF support layer significantly improved by 7 the water permeability and by 10° the hydrophilicity.^[20]^ On the same idea, Kang *et al*. fabricated novel PSF UF membrane using sulfonated graphene oxide (SGO) as additives. They showed that adding small amount (less than 3 wt %) of SGO improved wettability, porosity and mean pore size of the MMMs membranes compared to the raw ones. They also significantly enhanced the water flux of the membranes, as the ones prepared by adding 1.5 wt % SGO exhibited a 125 % higher water flux compared to the raw PSF membrane. The addition of SGO hydrophilic additives also showed better results in BSA rejection, 98 %, as well as better long‐term BSA separation performance.^[21]^ In addition, Alkhouzaam *et al*. investigated fouling resistance of PSF UF MMMs prepared using the phase inversion technique with low loadings of polydopamine‐functionalized GO particles (rGO‐PDA). The highest pure water permeability was obtained with 0.1 wt % of rGO‐PDA to be approximately twice that of the raw membrane. All membranes exhibited complete rejection of BSA and humic acid (HA), and showed almost similar performance against different dyes. The FRRs of the raw PSF after three fouling cycles against BSA and HA were recorded to be 57.8 % and 70.7 % respectively.^[22]^

A few studies focused on PSF HF MMMs based on GO but mostly for gas separation application.[^23^, ^24^, ^25^, ^26^] To the best of our knowledge, this study is the first one in addressing a variety of foulants with different natures and sizes using 1 wt % GO modified PSF HF MMM. Many studies focus selectively on a single type of foulant, this one would investigate performances and anti‐fouling properties of PSF/GO MMM not only on BSA but also on other foulants commonly found in wastewater. This study focused on the In here, GO was synthesized by Hummer′s method and inserted into PSF HF membranes made by Non‐solvent Induced Phase Separation (NIPS) process using a spinning line. The successful preparation and dispersion of GO and the modified membranes properties were presented in an unpublished previous study. The performances of the HF membranes for water purification application were characterized by water permeability and antifouling properties using several sized model molecules commonly found in water: BSA, humic acid (HA) which is virus‐like size, Oil emulsion and *E. coli* bacteria.

## 2 Materials and Methods

### 2.1 Materials

The chemicals employed for the preparation and characterization of HF PSF membranes were utilized in their as‐received state without further purification. Polysulfone (PSF) with a molecular weight of 75 kDa (CAS: 25135–6‐51‐7) and polyvinylpyrrolidone (PVP) with a molecular weight of 30 kDa (CAS: 9003–6‐39‐8) were generously provided by Polymem, a company specializing in such materials. The solvent N‐Methyl‐2‐pyrrolidone (NMP) of 99 % purity was sourced from Carlo Erba Reagents (CAS: 872–50‐4). Ethanol (CAS: 64–17‐5) and n‐hexane (CAS: 110–54‐3), also supplied by Carlo Erba Reagents, possessed a purity level exceeding 95 %. For evaluating fouling resistance, solutions of phosphate buffered saline (PBS) from Roth, bovine serum albumin (BSA) with a molecular weight of 67 kDa and a purity of ≥96 % from Sigma‐Aldrich, and humic acid (HA) with CAS: 1415–93‐6 from Sigma‐Aldrich were utilized. To gauge the oil‐in‐water separation efficiency of PSF HF membranes, the previously mentioned n‐hexane and sodium dodecyl sulfate (SDS) of ≥99 % purity (CAS: 151–21‐3) from Sigma‐Aldrich were employed. For bacterial filtration experiments, a ready‐to‐use Lysogeny Broth (LB) Miller culture medium from Sigma, France, was employed for growth and quantification purposes. The phosphate buffer, consisting of a water‐based salt solution with a concentration of 12.9 mM and a pH value of 7.0±0.1, was prepared using KH_2_PO_4_ at 1.06 g/L and Na_2_HPO_4_.12H_2_O at 4.34 g/L. B agar plates were produced by adding 15 g/L of microbiological agar from Sigma, France, into the LB solution. Throughout the experimental procedures, deionized water sourced from Millipore Milli‐Q was employed in all aqueous solutions.

### 2.2 Membranes Preparation

To initiate the process, polymer solutions were meticulously prepared. The initial solution was utilized for crafting the benchmark membrane, composed of NMP (59 wt %), PSF (22 wt %), and PVP (19 wt %). Depending on the designated mass, NMP was introduced into a dual‐walled glass reactor, where it was stirred and gradually heated to reach a temperature of 50 °C. Subsequently, the polymers were incrementally introduced and mixed until complete dissolution was achieved. In the case of the second solution, GO was first synthesized utilizing the Hummers method.^[28]^ This GO material, corresponding to wt. 1 % of PSF was then dispersed within NMP, which had been preheated to 50 °C, and subjected to ultrasonic treatment using a Bandelin electronic GmbH & Co UW 2200 ultrasound probe for a duration of 2 hours. 1 wt % GO was chosen in relation with polymer solution viscosity as presented in Figure S1, so that it is the closest to the one of the raw polymer solution, to ease the adjustments of spinning line process parameters. The resulting mixture was transferred to a dual‐walled glass reactor, stirred, and heated to 50 °C, after which the polymer components were added following the same procedure as mentioned earlier. On the day preceding the spinning process, the polymer solution along with the bore liquid–consisting of NMP and water–were introduced into the spinning line′s reactors. These solutions were degassed overnight using an ILMVAC GmbH vacuum pump. The fabrication of PSF HF membranes employed a spinning technique via the dry/wet phase inversion process. The polymer solution and bore liquid (composed of NMP and water) were extruded through a spinneret with dimensions of 1.1/0.7 mm. The extrusion took place with an air gap distance of 20 cm, leading to immersion in a coagulation bath containing soft water for solvent exchange. The membranes thus formed were thoroughly washed with water and collected at a rate of 9.6 m/min. Subsequently, the PSF HF membranes underwent a thorough wash in a 12.5 ppm chlorine solution for a period of 4 hours, followed by conditioning in a glycerin solution to prepare them for storage. For the sake of reference, the PSF HF membranes prepared without GO were denoted as “P,” while those incorporating GO were referred to as “PG”.

### 2.3 Characterizations

The membrane′s morphology, encompassing both its cross‐section and surface, was observed through the utilization of a Hitachi S4800 scanning electron microscopy system (SEM) after prior nitrogen cold‐cutting. The average pore sizes of the PSF HF membranes were deduced by employing Image J acquisition software to analyze SEM images. To delve into the surface morphology of the PSF HF membranes, atomic force microscopy (AFM) in tapping mode was conducted using an AFM Nano‐Observer from CSInstruments. During this process, silicon cantilevers (PPP‐NCH, Nanosensors) featuring a typical tip radius of 5 nm were employed. The cantilevers exhibited a resonance frequency of approximately 235 kHz. Assessment of the mechanical properties of both the unmodified and modified PSF HF membranes was carried out using a dynamic mechanical analysis device (Z005, 5 kN Proline, Zwick Roell). A 10 N sensor was employed, and a tensile testing speed of 0.4 mm min^−1^ was applied. The Young′s modulus of the membranes was directly calculated from the device′s measurements. Water contact angles (WCA) were measured utilizing a tensiometer/goniometer (ILMS GBX) equipped with an optic telecentric F55 double focal monochrome and GBX software. In this procedure, each sample was subjected to the deposition of approximately 0.5 μL of ultrapure water on the membrane′s surface using a fine needle.

### 2.4 Filtration Experiments

An I‐shaped hollow fiber bundle, consisting of 10 wet fibers measuring each 27 cm long (equivalent to an effective membrane area of 63.28 cm^2^), was prepared using a homemade polyvinyl chloride (PVC) housing module and an epoxy resin as sealing agent. The initial step involved the conditioning of the membrane through the application of a pressure of 1.5 bar using distilled water, a process that extended over a duration of 30 minutes. The measurement of pure water flux (J_w_) was conducted under ambient conditions through a frontal external‐internal dead‐end filtration approach for each individual membrane. This procedure involved the circulation of pure water across the membrane system, applying a range of applied pressures spanning from 0.5 to 1.5 bar. Each point was obtained measuring the mass passing through the membrane, from the outside to the inside of the membrane, during 20 minutes for each pressure step using an electronical scale. The calculation of pure water flux, denoted as J_w_ (L.h^−1^ m^−2^), was performed using the formula:

(1) $$Jw=\frac{Q}{A}\;\left(L\cdot h^{-1}\cdot m^{-2}\right)$$

**Figure 1:**
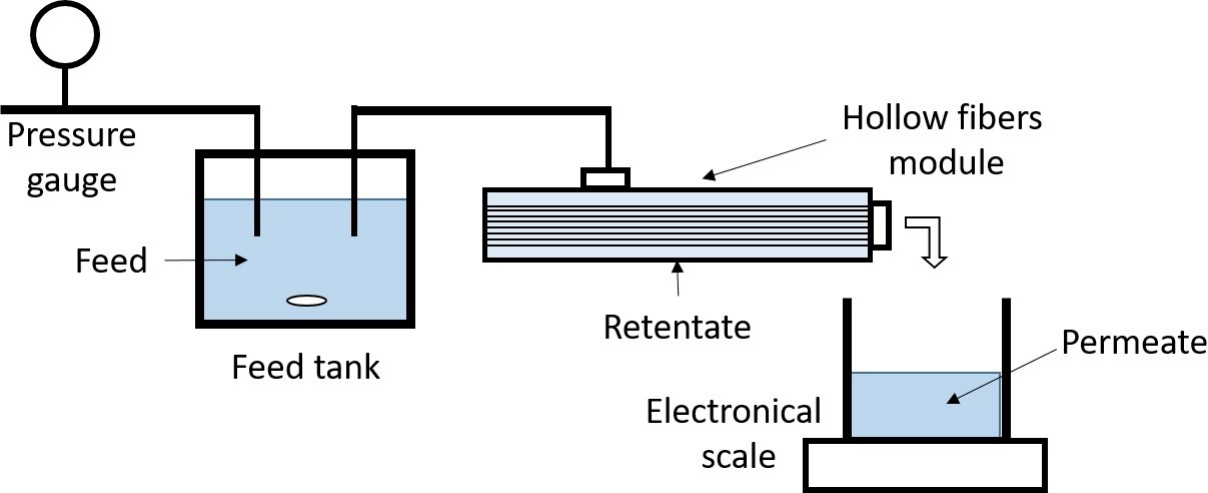
Hollow fiber dead‐end filtration system.

Here, Q (L h^−1^) represents the volume of water that traversed the membrane, and A (m^2^) is the membrane′s surface area. The permeability was ascertained by analyzing the slope of the linear relationship between JW and the applied pressure. An illustration of the filtration system employed is provided in Figure 1.

Antifouling properties were studied with diverse molecules presented in Table 1. Rejection trials were carried out with various molecules, including BSA and HA, to assess the anti‐fouling characteristics of the PSF HF membranes. The concentrations of fouling agents in both the feed solution (C_f_) and the permeate solution (C_p_) were determined through flow injection analysis utilizing a refractometer 2414 from Waters Corporation. The foulant rejection (R) was calculated using the subsequent formula:

(2) $$R=\left(1-\frac{C_{p}}{C_{f}}\right)\times 100\;\left(\%\right)$$

**Table 1:** The dimensions of fouling particles (particle sizes were ascertained using dynamic light scattering (DLS)).

Object	BSA	HA	Oil droplets	Bacteria
Size (nm)	10	400	500–1500	600–4000^[27]^

The protein solution was created by dissolving 2 g of BSA in 2 L of PBS solution at a pH of 7.4. Prior to experimentation, the membrane underwent initial conditioning through the application of a pressure of 1.5 bar using distilled water, a process that lasted for 30 minutes. Initial external‐internal water filtration of the membrane is then carried out for 20 min at 0.5 bar and the steady pure water flux was recorded (Jw_0_) using a balance and data acquisition software. The tank was then filled with the BSA solution and filtration was carried out at 0.5 bar for 2 hours and foulant flux was recorded (Jw_f_). The BSA was removed by mechanical agitation and then backwashing with water applying a pressure of 1.5 bar internally‐externally for 5 min. Finally, a last external‐internal filtration cycle with water for 20 min at 0.5 bar was performed and the steady flux was recorded (Jw_1_). The values for the total fouling ratio (R_t_), flux recovery ratio (FRR), reversible fouling ratio (R_r_), and irreversible fouling ratio (R_ir_) were computed utilizing Equations (3) through (6), respectively^[29]^:

(3) $$R_{t}=\frac{J_{w_{0}}-J_{w_{f}}}{J_{w_{0}}}\times 100\;\left(\%\right)$$

(4) $$FRR=\;\frac{J_{w_{1}}}{J_{w_{0}}}\times 100\;\left(\%\right)$$

(5) $$R_{r}=\;\frac{J_{w_{1}}-J_{w_{f}}}{J_{w_{0}}}\times 100\;\left(\%\right)$$

(6) $$R_{ir}=\;\frac{J_{w_{0}}-J_{w_{1}}}{J_{w_{0}}}\times 100\;\left(\%\right)$$

The feed foulant solution, foulant retentate and permeate (Figure 1) concentrations are calculated from Beer Lambert′s Law and an ultraviolet (UV) spectrometer on the absorbance at 280 nm (Uviline Connect Series 940). The foulant rejection was determined using Equation (2), in which C_p_ and C_f_ represent the concentrations of the foulant in the permeate and feed, respectively.

The same protocol was applied for HA adsorption using a 20 ppm concentration into PBS solution and recording the absorbance at 254 nm.

The preparation of Oil‐in‐Water emulsions and filtration protocol were based on literature[^30^, ^31^] and on the above equations. The feed foulant solution, foulant retentate and permeate concentrations are once again calculated using the UV spectrometer on the absorbance at 225 nm. The sizing of emulsion droplets within the oil‐in‐water (O/W) emulsion was assessed through dynamic light scattering (DLS) using the Litesizer 500 instrument from Anton Paar.

### 2.5 Bacterial Removals

#### 2.5.1 Preparation of the Bacterial Suspension

For the retention assessments, a non‐pathogenic Gram‐negative bacterium, Escherichia coli (K12 DSM 423, sourced from DSMZ, Germany), was employed. To facilitate the growth and enumeration of the bacteria, a ready‐to‐use Lysogeny Broth (LB) Miller culture medium obtained from Sigma, France, was utilized. The bacterial cultures were initiated from cryopreserved portions of *E. coli* stored at a temperature of −20 °C. These aliquots were introduced into fresh LB medium (at a volume ratio of 4 % v/v) and subsequently cultivated for 18 hours at a temperature of 37 °C under continuous agitation (at 110 rpm). This cultivation allowed the bacterial culture to enter the stationary phase, characterized by a stable optical density at 600 nm (OD600 nm) of approximately 5. This optical density corresponds to an approximate bacterial count of 10^9^ colony‐forming units per milliliter (CFU/mL).^[32]^ Subsequently, the bacterial cultures underwent centrifugation at 4500 rpm and 4 °C for a duration of 12 minutes. The LB supernatants were discarded, and the resulting pellets were re‐suspended in an equivalent volume of phosphate buffer. This step was taken to prevent any potential bacterial proliferation. Following this, the bacterial suspensions were further diluted in phosphate buffer to achieve a concentration of approximately 5*10^7^ colony‐forming units per milliliter (CFU/mL). These resulting bacterial suspensions were then utilized for both the filtration experiments and the subsequent plate counting procedures.

#### 2.5.2 Bacterial Counting

A conventional plate assay method was used to enumerate bacteria in the feed, in the permeate and in the retentate.^[32]^ Following the completion of the above steps, all the plates were placed in an incubator set at 37 °C for a period of 48 hours. Once viable bacteria had proliferated on the plates, the colonies were enumerated using plates that had been appropriately diluted. It is important to note that each colony on the plates originated from a single initial bacterium. Bacterial concentrations in the samples were calculated as the average colony number divided by the inoculation volume (i. e., 200 μL). Each count was triplicated. Quantification limits were comprised between 3 CFU/mL (lower limit) and 150 CFU/mL (upper limit), where CFU means Colony‐Forming Unit. Negative controls (*i. e*. without bacteria) were always run in parallel to check the sterility. Bacterial removal was expressed either in log‐removal value (LRV in log) or in a reduction rate (Re in %). LRV was calculated according to Equation 7:

(7) $$LRV=\;{log}_{10}\left(\frac{C_{f}}{C_{p}}\right)\;\left(log\right)$$

Here, C_f_ (CFU/mL) denoted the bacterial concentration present in the feed used during the filtration experiments (Figure 1), while C_p_ (CFU/mL) represented the bacterial concentration detected in the permeate after filtration. It is noteworthy that a higher retention of bacteria by the membrane corresponded to a reduction in the Log Reduction Value (LRV).

The calculation of the reduction rate is achieved through the application of the subsequent equation:

(8) $$Re=\;\frac{\left(C_{f}-C_{p}\right)}{C_{f}}\times 100\;\left(\%\right)$$

The formula to obtain Re from LRV is given by Equation 9.

(9) $$Re=\left(1-10^{-LRV}\right)\times 100\;\left(\%\right)$$

The content Δads of bacteria that was assumed to be adsorbed onto the membranes at the end of each filtration step was determined as follows:

(10) $$\;\Delta ads=\frac{\left(n_{f}-n_{p}-n_{r}\right)}{n_{f}}\;\times 100\;\left(\%\right)\;$$

Where n_f_ is the bacteria quantity filtrated (CFU), n_p_ is the bacteria quantity in the permeate at the end of the filtration (CFU) and n_r_ is the quantity of bacteria that was in suspension in the retentate at the end of filtration (CFU).

#### 2.5.3 Filtration of the Bacterial Suspension

Filtrations were carried out in the filtration modules as explained above (Figure 1), the whole filtration system, including membrane, was disinfected with ethanol (70 % *v/v* in water) before each bacteria filtration. The system was then rinsed with sterile ultrapure water (with a water volume that was at least 2 times the total liquid volume of the filtration system). The initial membrane flux was measured with water then a bacterial suspension at about 5x10^7^ CFU/mL, prepared as mentioned in Section 2.5.1 was filtrated. The bacterial concentration was quantified within the suspension utilized as the initial feed prior to filtration. The filtration process was carried out in a dead‐end mode, maintaining a constant pressure of 1 bar. The flux of the permeate was measured by employing a balance in conjunction with data acquisition software. The air used to compress the bacterial feed in the feed tank was sterilized by a hydrophobic filter with a 0.45 μm mean pore diameter (Sartorius, France). At the end of the filtration, the bacteria concentration was measured again in feed solution bust also in the permeate and the retentate by the plate assay method. Deionized water backwashing step was carried out with a pressure of 1.5 bar internally‐externally for 5 min and the water fluxes were measured on the backwashed membranes as explained in previous section until the flux reached a plateau. The relative rate of flux densities loss (PF) was assessed using Equation 11.

(11) $$PF=\;\frac{\left(J_{w_{0}}-J_{w_{f}}\right)}{J_{w_{0}}}\times 100\;\left(\%\right)\;$$

Where Jw_0_ (L.h^−1^ m^−2^) was the flux density initially measured and Jw_f_ (L.h^−1^ m^−2^) the flux densities measured either at the end of the filtration or after the washing steps. The fouling was considered to be totally reversible when PF was zero and irreversible (partially or totally) when PF was different from zero. This PF calculated for bacteria filtration could refer in terms of calculation to the R_ir_ presented above.

## 3 Results and Discussion

The aim of this study is to investigate the fouling propensity of PSF HF MMMs modified with 1.0 wt % GO based on a range of model contaminants from BSA to HA including *E. coli* bacteria and oil‐in‐water emulsion. First, the structural and physico‐chemical properties of these GO‐modified membranes will be characterized. Then, their fouling properties will be detailed for all four contaminants and finally compared and discussed based on literature.

### 3.1 Structural and Physico‐Chemical Characterizations

The successful synthesis of GO using Hummers’ method and its good dispersion in the polymer matrix was confirmed respectively by XRD and FTIR analyses in a previous study.^[33]^ An increase of interlayer distance, from 3.34 Å to 6.6 Å, was observed and explained by the intercalation of water molecules between the oxidized graphene layers. In fact, the diffraction peak attributed to the (002) plane of graphite, initially at 2θ=26.6°, shifted to 2θ=13.4° after the introduction of oxygen functionalities as shown in Figure S2. On Figure S3, all the notable signals attributed to the PSF aromatic ring, the SO₂ group and the symmetric and aromatic ether bond can be observed. In our case, any significant difference was observable in PG spectrum by the addition of GO supposedly due to the too low amount of powder dispersed in the polymeric matrix.

**Figure 2:**
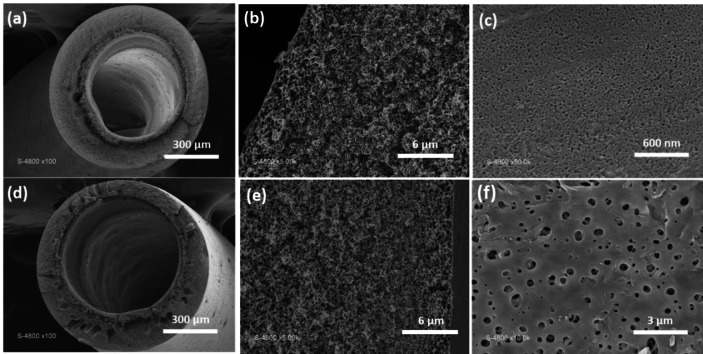
Scanning electron microscopy (SEM) images depicted cross‐sectional views (a), zoomed (b) and surface characteristics (c) of the P membranes, along with cross‐sectional views (d), zoomed (e) and surface features (f) of the PG PSF HF membranes.

In Figure 2 and Table 1 cross‐sectional and surface SEM images of the PSF HF membranes are displayed. Each membrane displayed a porous selective top‐surface and a cross‐section with completely sponge‐like asymmetric structure with randomly dispersed voids. In fact, the rapid out‐diffusion of solvent caused the instantaneous solidification of the external surface of the membrane when the polymer solution is immersed into the coagulation bath. Subsequently, the non‐solvent (water) permeated the membrane, leading to the formation of finger‐like pores that extended downward from the surface. This phenomenon is more prominent in the modified membranes, as illustrated in the cross‐sectional SEM images. Due to its multiple hydrophilic groups present on the surface, GO display great affinity with water which increased the mass transfer rate between the non‐solvent (water) and the solvent (NMP) during the phase inversion process allowing larger pore channels to grow inside the membrane.^[34]^ During phase inversion process, the demixing phenomenon between NMP and water slowed down due to the increasing presence of the solidified polymer. This process partially obstructs the separation of components and contributes to the creation of a sponge‐like cellular pore structure.^[35]^

**Figure 3:**
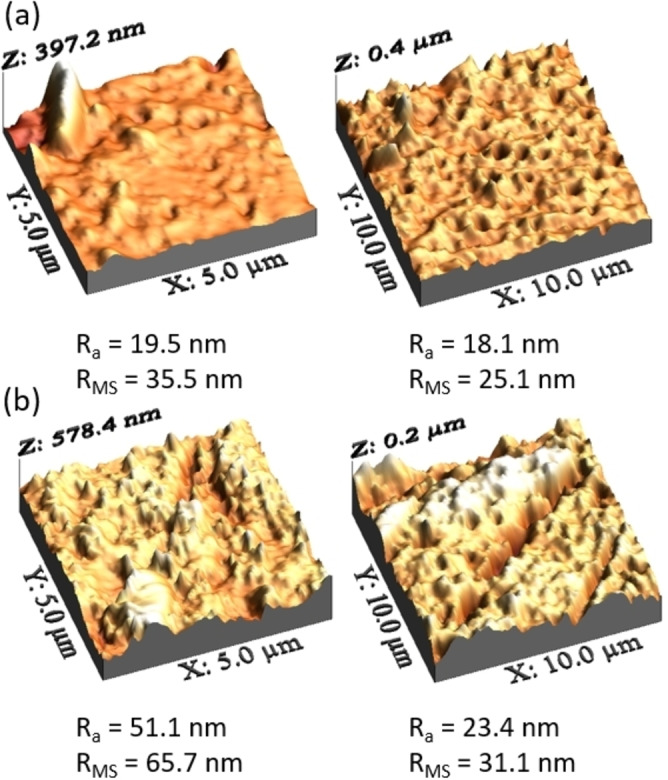
Three‐dimensional AFM images were obtained for the untreated PSF HF membranes (a) and the PSF HF membranes modified with GO (b). These images were captured across areas measuring 5×5 μm and 10×10 μm. Additionally, the root mean square roughness (R_MS_) and average roughness (R_a_) values were computed for these surfaces.

Figure 3 displays the three‐dimensional surface AFM images of both the untreated and GO‐modified PSF HF membranes. These images were captured using scan sizes of 5×5 μm and 10×10 μm, and the corresponding roughness average (R_a_) and root‐mean‐squared roughness (R_MS_) values were calculated. The AFM images of the membranes revealed a surface pattern characterized by ridges and valleys. In these images, the brighter regions corresponded to the membrane′s highest points, while the darker regions represented its lowest points or pores. It can be understood that the surface roughness increased with the addition of GO which is consistent with other studies.[^36^, ^37^, ^38^, ^39^] The escalation in surface roughness can be linked to the augmented pore size evident in the preceding SEM images. Moreover, this phenomenon is attributed to the rapid interaction between solvent and non‐solvent during the phase inversion process, facilitated by the hydrophilic properties of GO. The swift solvent exchange might lead to the creation of polymer spheres or nodules on the membrane surface, thereby contributing to an increased roughness. Alihemati *et al*. studied the preparation of a double‐skinned thin film composite and thin film nanocomposite HF membrane with GO addition on the outer layer. They showed a R_a_ increase from 16.7 nm to 23.6 nm with only 0.1 wt % GO addition.^[26]^ Contrary observations have been reported in various studies, suggesting a different trend in surface roughness when incorporating GO. Some of these studies advocate for membranes exhibiting smoother surfaces and higher porosity, primarily driven by the intention to mitigate fouling issues effectively.[^37^, ^40^] It is also highly supported that a rough surface is desirable to have an increased water flux.[^41^, ^42^] However, a more detailed scrutiny of the images acquired through AFM analysis unveils the presence of distinct striations on the surface of the membrane. This can be explained by the orientation of the GO nanosheets caused by the passage of the polymer solution through the spinning nozzle.

Raw and GO PSF HF membranes were tested in terms of mechanical properties, hydrophilicity and water permeability in a previous unpublished study and the results are gathered in Table 2. Notably, despite the existence of several voids, the incorporation of a relatively small amount of GO into the PG membranes appears to safeguard their mechanical properties when compared to the untreated P membranes. This trend aligns with findings from numerous other studies.[^43^, ^44^] Continuing to introduce more GO into the polymer matrix might result in a reduction of both the tensile modulus and tensile strength, causing them to fall below the values observed in the untreated PSF. Pushing beyond a 1 %wt. GO content could potentially lead to adverse effects, particularly due to inadequate dispersion. Such poor dispersion could concentrate stress within the material, ultimately compromising its mechanical properties.^[19]^

**Table 2:** Characteristics of both the untreated and GO‐modified PSF HF membranes (with the error denoting the standard deviation derived from multiple samples).

	P	PG
Outer diameter (mm)	0.94±0.02	0.98±0.02
Inner diameter (mm)	0.53±0.02	0.67±0.02
Average pore size (nm)	19.3±1.5	263.4±75.6
Young Modulus (MPa)	130±5	144±9
Strength at break (N)	1.8±0.1	1.5±0.4
WCA (°)	100.1±3.5	92.4±2.3
Permeability (L.h^−1^ m^−2^ bar^−1^)	33±3	142±14

Fouling of membranes and the water permeability of membranes are greatly impacted by factors such as pore size and surface roughness. However, the hydrophilic nature of the surface also plays a significant role. The water contact angles (WCA) of untreated and graphene oxide (GO) modified PSF HF membranes were measured and are detailed in Table 2. The insertion of GO onto the PSF HF membrane enhanced its surface hydrophilicity as suggested in literature,[^14^, ^34^, ^38^, ^39^, ^44^] reducing WCA from 8 % for a 1 %wt. of GO. In contrast, Zhao *et al*. fabricated PVDF/GO UF membranes using an immersion precipitation phase inversion method, observing an 11 % reduction in water contact angle (WCA) with the incorporation of 1 wt % of GO.^[40]^ Once again, the controlled amount of GO is a key parameter to improve membranes properties as a too high content would damage mechanical properties but also affect hydrophilicity. Leaper *et al*. and Kang *et al*. who incorporated GO onto PVDF and PSF membranes respectively experienced this observation. They noticed that the decreasing WCA when adding GO would increase again when going to 2 wt % GO. Indeed, this phenomenon can be elucidated by the decrease in porosity and the aggregation of additives, leading to a decrease in the presence of effective hydrophilic functional groups on the membrane surface, along with the obstruction of membrane pores.[^45^, ^46^]

In accordance with surface hydrophilicity and pore size increase, the water permeability of GO modified PSF HF membranes was also improved. As presented in Table 2, the permeability was around 30 L.h^−1^ m^−2^ bar^−1^ for raw membrane P and was even 4 times higher for PG. Once more, this is attributable to the hydrophilic active sites on the GO surface, which act as donors, thereby enhancing porosity and the membrane′s capacity to absorb water, facilitating smoother water passage. This observation aligns well with what has been reported on the incorporation of GO into polymeric membranes.[^38^, ^45^, ^47^]

### 3.2 Anti‐Fouling Performances

#### 3.2.1 BSA

**Figure 4:**
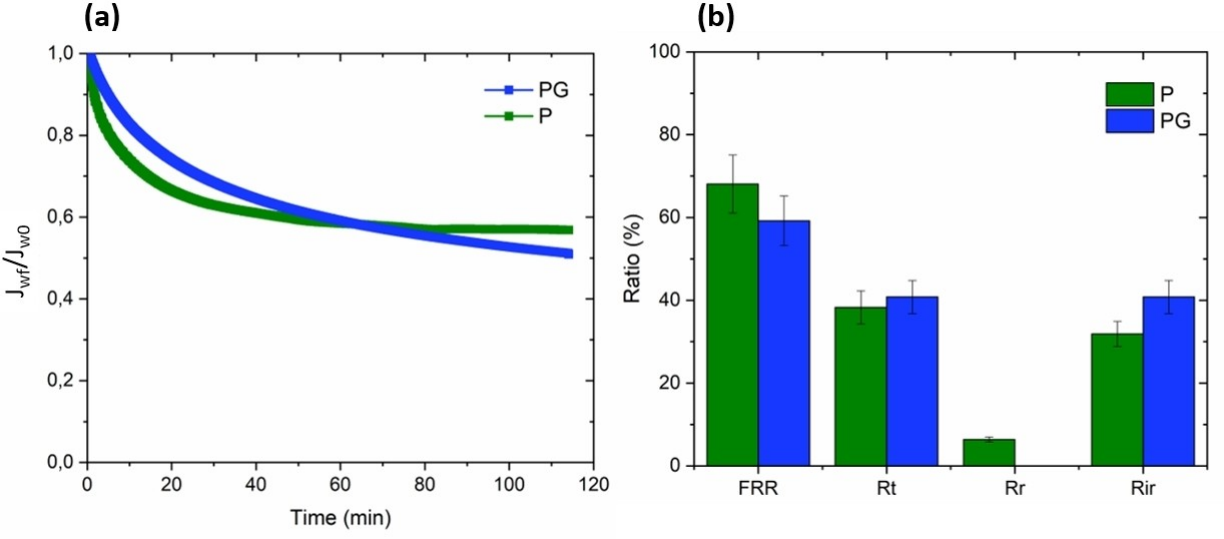
Normalized flux ratio over time (a) and fouling parameters (b) of raw and GO modified PSF HF membranes during BSA filtration (the error referred to standard deviation of 3 samples). BSA feed solution concentration is 1 g/L.

Initially, an organic compound of macro size, the BSA protein, was utilized to evaluate the fouling resistance of both untreated and GO‐modified PSF HF membranes. The size of BSA molecules was determined to be approximately 10 nm through dynamic light scattering (DLS) measurements. Figure 4 presents the normalized flux ratio over time along with the fouling parameters of BSA filtration for P and PG membranes.

Both membranes presented a decreasing flux ratio over time. After 120 min, stabilization is reached for P membrane with a flux reduction of about 42 % in contrast to PG where the flux still seems to be decreasing. Taking the standard deviation into account, the fouling parameters of raw and GO modified PSF HF membranes were quite similar with a FRR around 60 % and R_t_ and R_ir_ around 40 %. The inclusion of GO, which reduces the membrane′s hydrophobicity, is expected to enhance the rejection of BSA, a trend that has been documented in the existing literature.[^29^, ^48^, ^49^, ^50^, ^51^, ^52^, ^53^] In fact, the heightened hydrophilicity of the surface leads to greater interactions between the membrane surface and water molecules. This interaction hinders the bonding between the surface and foulants, subsequently diminishing the accumulation of these substances on both the membrane surface and within its pores. BSA is known to be hydrophobic in nature and should be less retained by GO modified than raw PSF HF membranes. However, according to Figure 4 it can be assumed that despite a lower WCA, BSA molecules attached the membrane surface and blocked the pores. Discussing about the fouling mechanism is challenging as it is very specific to the membrane nature, foulant nature and environment. However, on Figure 4, in the case of P membranes the flux decreases until attaining a bearing. As the pores are way smaller than PG membranes, we could imagine a cake layer formation on the surface of the membranes. In the contrary, for PG membranes the flux is still decreasing along time which may correspond better to an intermediate blocking of the pores by the proteins molecules.^[54]^ This phenomenon can be rationalized by the increased pore size and surface roughness found in GO‐modified PSF HF membranes. These characteristics, in turn, facilitate the movement of protein molecules into the matrix and promote the adsorption of foulants.^[3]^ In fact, pore size and surface roughness increase of PG prevailed over any improvements explaining why the incorporation of GO did not allow better BSA anti fouling performances. It should be noted that both pore size distribution and surface roughness result to a certain extent from the hollow‐fiber membrane spinning process. Nevertheless, it is to highlight that anti‐fouling performances of GO modified PSF HF membranes were still not altered compared to raw ones.

#### 3.2.2 Humic Acid

Fouling propensity of the as‐prepared membranes was then investigated by an organic acid foulant, HA solution, which size is around 400 nm as presented in Table 1.

**Figure 5:**
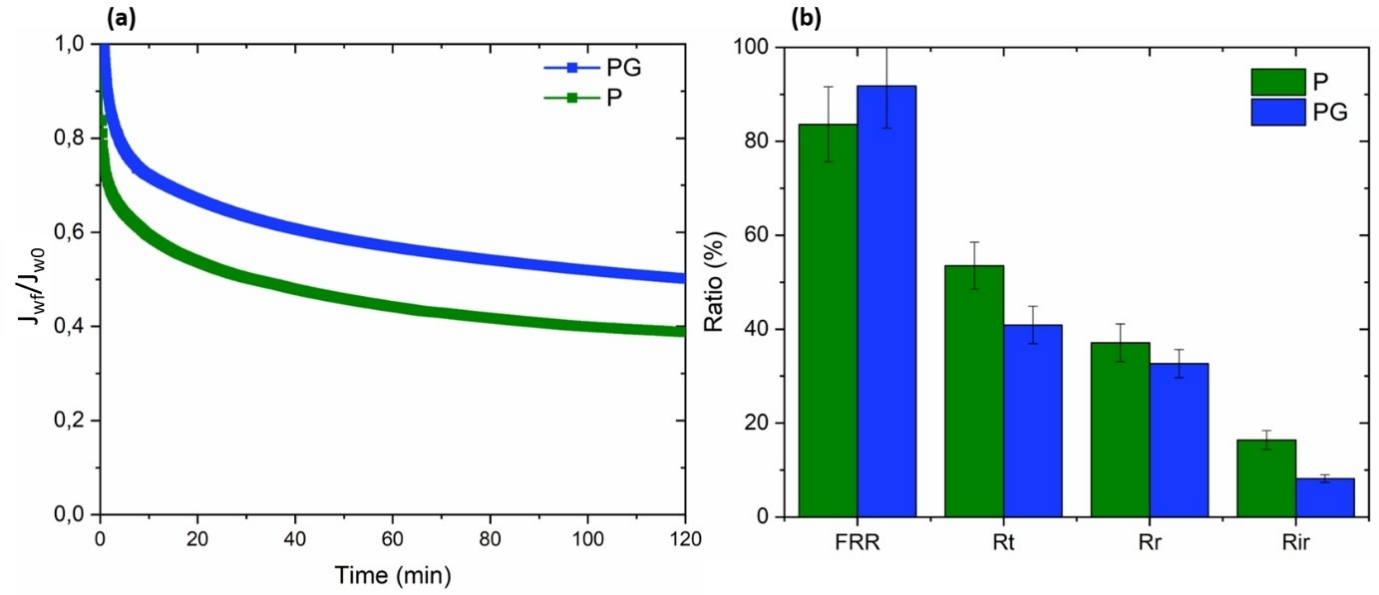
Normalized flux ratio over time (a) and fouling parameters (b) of raw and GO modified PSF HF membranes during HA filtration (the error referred to standard deviation of 3 samples). HA feed solution concentration is 0.2 g/L.

Both membranes showed in Figure 5a a similar flux trend, the flow initially decreased until it reached after 120 min a relatively stable level. Interestingly, the GO modified PSF HF membranes maintained a consistently lower flux loss, implying that the performance of the membranes was improved after inserting GO. These results can again be explained by changes in the WCA that could also significantly affect the performance of the membrane. To confirm the enhanced efficacy of the PSF HF membranes modified with GO, Figure 5b presents the determination of the flux recovery ratio (FRR) subsequent to cleaning the membrane surface with distilled water, as well as the comprehensive fouling ratio (R_t_), which encompasses both reversible fouling (R_r_) and irreversible fouling (R_ir_). Indeed, a higher FRR was observed for PG compared to P. PG also exhibited a lower R_t_ with an interestingly decreased R_r_. A reducing Rr means that HA adsorbed reversibly was removed after water backwashing, which is a good result as membranes lifetime is threatened by fouling and most of all irreversible fouling and chemical backwashing needed to clean them. R_ir_ initially at 16.4 % for P was reduced by half when adding GO. This shows that with GO, the PSF HF membranes are less sensitive to HA fouling and would have a longer lifetime. These are good results supported by the higher pore size and hydrophilic character keeping in mind that higher surface roughness of GO modified PSF HF membranes would tend to create affinity with foulants and so decrease fouling resistance.^[53]^ Once again, discussing about the fouling mechanism is challenging but here the behavior of P and PG membranes fluxes is similar. In fact, HA molecules are 40 times bigger than BSA ones, and they are bigger than the average pore size of both membranes. In that case, we could suppose cake layer formation and pore plugging.^[54]^

#### 3.2.3 Oil in Water Emulsion

**Figure 6:**
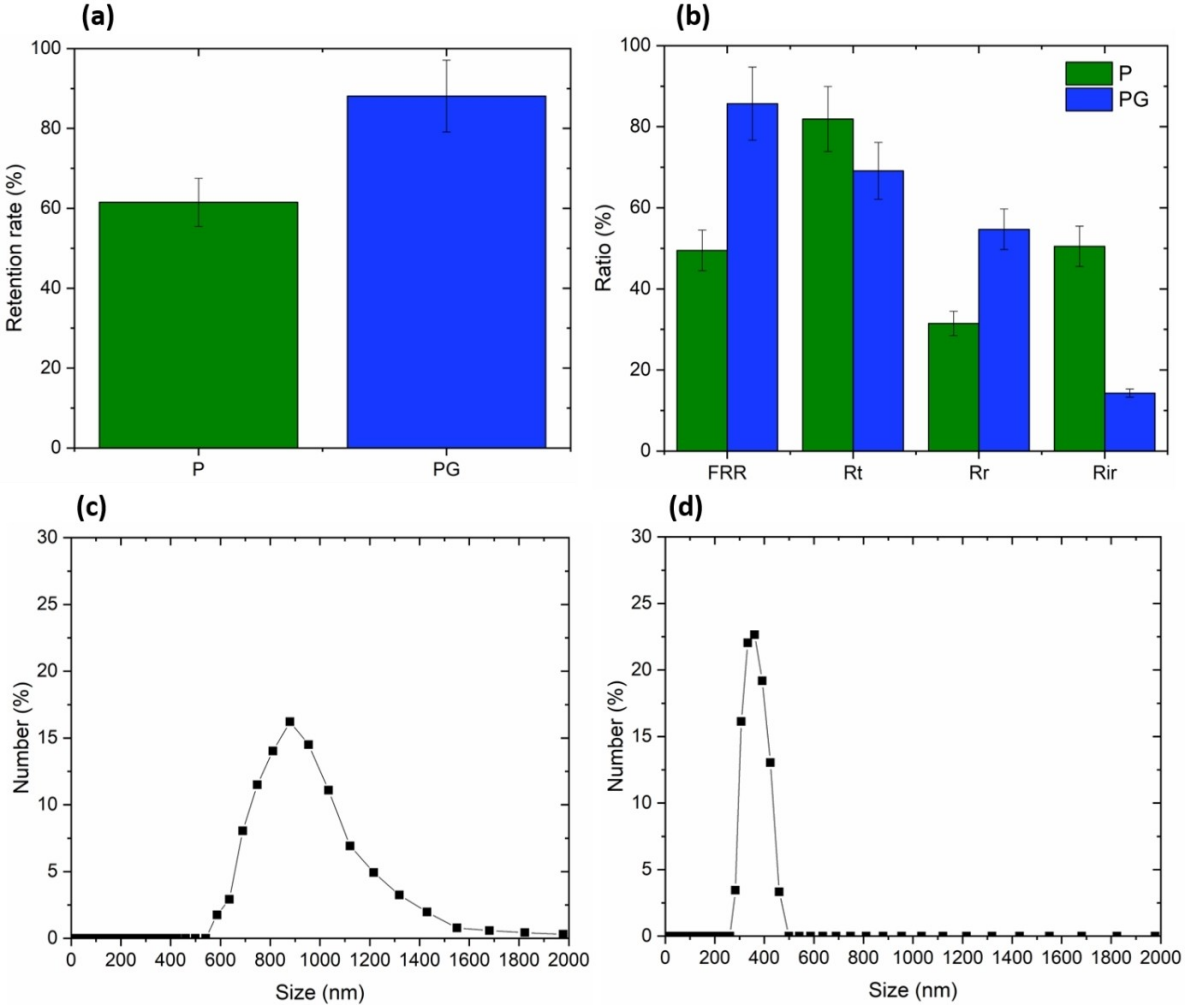
The retention rate (a) and fouling characteristics (b) of both untreated and GO‐modified PSF HF membranes, along with the distribution of oil droplet sizes in both the (c) feed and (d) permeate, are presented. The error bars represent the standard deviation calculated from three samples. The concentration of the oil‐in‐water feed solution is 66.5 g/L.

Subsequently, an emulsion solution containing oil dispersed in water was created to assess the separation effectiveness of the PSF HF membranes. The outcomes of this evaluation are depicted in Figure 6. GO modified PSF HF membranes exhibited great separation performance compared to raw PSF HF membrane by increasing the oil retention rate from 60 to 90 % as shown in Figure 6a. From Figure 6b. it was noticed that PG recovered almost 90 % of water flux after emulsion filtration against 50 % for P. In addition, R_t_ was reduced by 16 % and mostly converted into reversible fouling lowering the irreversible fouling R_ir_ from 50 to almost 10 %. Figures 6c and d display the distribution of oil droplet sizes within the emulsion for both the feed and permeate. In the feed, the oil droplet sizes ranged from 500 to 1500 nm, whereas in the permeate, the sizes ranged from 300 to 500 nm. These findings are promising, drawing parallels with the observations made by Modi *et al*., who noted comparable exceptional antifouling attributes, achieving a flux recovery of 90.5 % and notably high oil/water separation (98.7±1.2 %) using PSF composite HFMs incorporating a 1 wt % combination of CNTs and GO nanohybrids.^[56]^

#### 3.2.4 *E‐coli* Bacteria

**Figure 7:**
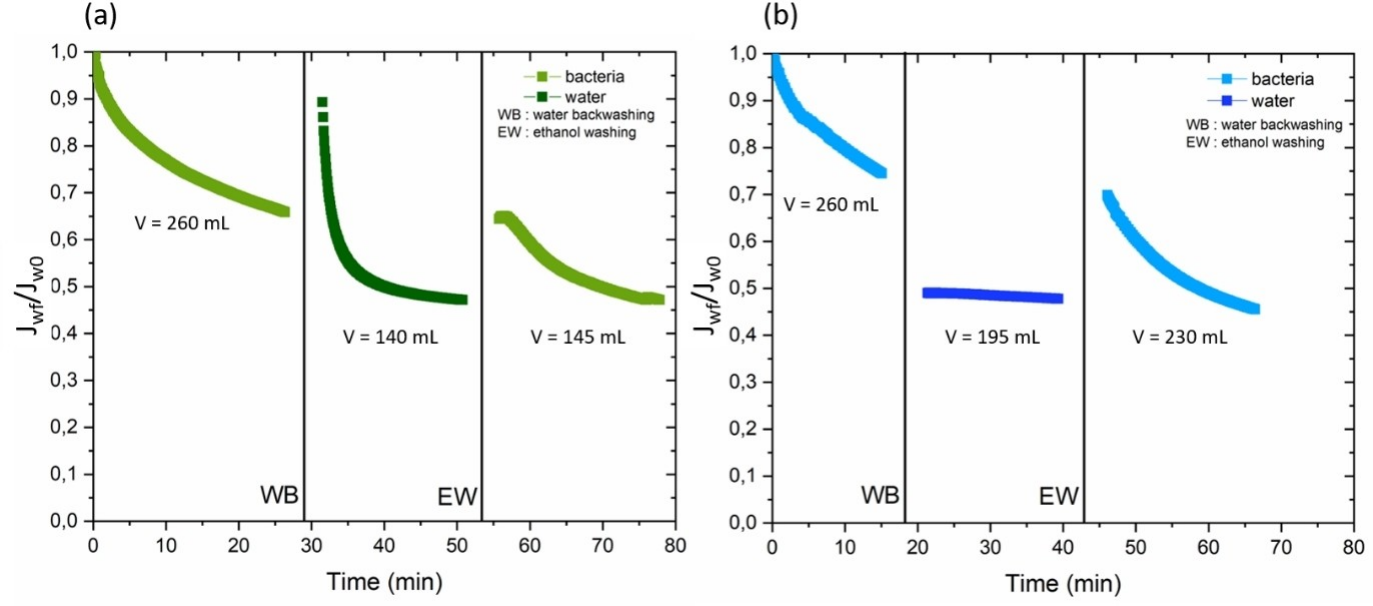
Normalized flux for bacteria filtration of raw (a) and GO modified PSF HF membrane (b). J0 (L/h.m^2^) is the initial water flux of the sterilized modules at 1 bar (*i. e*. 95±10 L.h^−1^ m^−2^ for the P module and 160±10 L.h^−1^ m^−2^ for the PG module). J (L.h^−1^ m^−2^) designates the water flux measured with time. The filtrated volumes V (mL) are the volumes respectively filtrated at each step.

Finally, the retention performance of raw and GO‐modified PSF HF membranes toward *E. coli* was investigated. A module of raw PSF HF membranes (P) and a module of GO‐modified PSF HF membranes (PG) were used respectively for the filtration tests. Filtration tests first consisted in filtrating 270±20 mL of bacterial suspension. Membranes were then backwhashed with water (Section 2.5.3) and sterilized once again with an ethanol 70 % *v/v* washing (Section 2.5.3). A second bacteria filtration was therafter carried out. Results are presented in Figure 7 and Table 3.

**Table 3:** Flux and retention performances for raw membranes (P) and GO PSF HF membranes (PG): **A**. Relative rate PF (%) of flux densities loss for each filtration step: first bacteria filtration (PF_1_), after water backwashing (PF_WB_), after ethanol washing (PF_EW_) and finally after the second bacteria filtration (PF_2_); the initial flux value was fixed to J_0_ in all cases and the final value was the last flux measured during the considered filtration step. **B**. Log Removal Values (−) and contents Δads (%) of bacteria that were assumed to be adsorbed onto the membranes at the end of each filtration.

**A**	PF_1_ (%)	PF_WB_ (%)	PF_EW_ (%)	PF_2_ (%)
P	35±4	50±5	35±4	50±5
PG	25±3	50±5	30±3	55±6

B	First filtration		Second filtration	
	LRV (log)	Δads (%)	LRV (log)	Δads (%)
**P**	−6.6±0.6	61±6	−5.1±0.5	100
**PG**	−5.3±0.5	55±6	−4.2±0.4	78±12

Finally, LRV obtained for raw (P) and GO‐modified PSF HF (PG) membranes were all higher than a log reduction of 4 (Table 3B) regardless of the bacteria filtration, meaning that the membranes retained at least 99.9999 % (Re). These LRV are significant because they are superior to 1 log which is the minimum retention recognized by the World Health Organization to be representative of a retention performance and close to −6 which is the LRV recommended by Food and Drugs Administration (FDA) for water disinfection treatment against bacteria.^[57]^ Bacteria size are usually of about 0.6–4 μm.^[27]^ Although P membranes exhibited better LRV than PG ones (around −1 log more, Table 3A), due to a much smaller mean pore size diameter as referred in Table 2 (20 nm for P membranes vs 260 nm for PG membranes), the two membranes have nevertheless reduction rates (Re) that are both nearly 100 % (i. e. 99.999999 % for P vs 99.99999 % for PG for the first filtration, Table 3A). The retention performances of P and PG membranes could be explained by a simple steric phenomenon. Nonetheless, it was also hypothesized that bacterial adsorption onto and within the membranes might play a role in achieving the notable retention performances. This assumption is consistent with the significant decrease of normalized flux during filtration time (Figure 7) and the mass balance on bacteria showing high adsorption contents Δads onto the membranes (Table 3B).

Figure 7 shows that flux had a same behavior for both P and PG membranes. It indeed decreased at each filtration step, in the same proportions since the associated PF values are quite similar for both P and PG membranes (Table 3A). However, filtration is nearly 2‐times faster with GO (Figure 7) which is logically related to the higher porosity and higher initial water flux J0. The flux exhibited a decline while undergoing bacteria filtration, a phenomenon that could be attributed to the occurrence of membrane fouling triggered by the presence of bacteria. Contrary to what might be expected, water backwashing (WB) did not allow the membranes to recover their initial water flux J0 and it significantly decreased the PF values (PF_WB_ from 30 % to 50 % on average, Table 3A). An explanation could be that WB pushed the adsorbed bacteria deeper inside the membrane porosity. An ethanol washing (EW) helped to recover a flux similar to the last one obtained during the first bacteria filtration (Figure 7 and PF_EW_ in Table 3A). Ethanol chemical action was likely necessary to kill part of the adsorbed bacteria. However, a higher contact time with ethanol should have been more benefic to flux recovery. The flux still continued decreasing during the second bacteria filtration, reaching final PF values of about 50 % (PF_2_, Table 3A). It could be observed that Δads increased while LRV decreased (Table 3B). These latter results could origin from a higher number of bacteria located on and inside the membranes, compared to the first bacteria filtration.

#### 3.2.5 Discussion

This section aims to elucidate and contrast the behavior of PSF HF membranes modified with GO in response to various types of foulants. Table 4 summarizes all fouling parameters of both raw and GO modified PSF HF membranes for BSA, HA, oil‐in‐water and *E. coli* foulants filtration. Koo et al. conducted a review focusing on the influence of specific physicochemical factors on the tendency for membrane fouling, utilizing fouling indices as a basis for their analysis. They explained that several factors affect membranes fouling properties such as membranes geometry, hydrophilicity, roughness or charge, feedwater composition or hydrodynamic conditions. They emphasized that, as a general trend, membranes exhibiting hydrophilic properties that encourage water adsorption tend to be less prone to fouling when compared to hydrophobic membranes. The size of membrane pores plays a substantial role in determining its vulnerability to fouling, given that membranes with larger pore sizes are more likely to facilitate the penetration of molecules into the matrix. This can result in the accumulation of molecules and subsequent blockage of the pores. Finally, higher surface roughness tends to enhance foulant adsorption on membrane surface creating also a cake layer blocking pores and reducing water flux.^[58]^ Das *et al*. studied advances in anti‐fouling membranes to improve process economics and sustainability of water treatment. They evaluated that fouling exhibited a high degree of specificity, manifesting as a complex phenomenon contingent on the unique combination of present foulants, their concentrations, and the intricate interactions unfolding between the foulants and the particular membrane being employed. They also emphasized that characteristics inherent to the membrane, including hydrophilicity, surface charge, and surface roughness, wielded a significant influence on the membrane′s susceptibility to fouling.^[59]^ Alihemati *et al*. emphasized that as time progresses, the water flux experiences a swift reduction owing to the interaction between the porous membrane surface and the feed solution, coupled with the entrapment of foulants within the substrate pores. However, they noted that the inclination for fouling in the membranes diminishes with an increase in the proportion of GO within the active layer. This increase in GO content leads to a decrease in water contact angle (WCA) and subsequently curbs the membranes’ tendency to foul.^[26]^ In our study, PSF HF membranes modified with GO exhibited enlarged pore sizes and heightened surface roughness, accompanied by a decreased hydrophobic nature. Although we did not assess surface charge, previous literature indicates that the inclusion of GO might lead to the creation of a hydration layer on the membrane surface. This hydration layer, formed through the development of a hydration shell, has the potential to hinder the passage of hydrophobic molecules through the modified membranes.^[60]^ Nevertheless, the water contact angle (WCA) measurement of the PG membrane still registers above 90°. It has been documented that PSF membranes carry a negative charge, boasting a zeta potential of approximately −10 mV at a pH of 7.4.^[61]^ Furthermore, owing to its inherent functional groups, GO also holds a negative charge.^[62]^ Hence, the incorporation of GO would further enhance the negative charge of the PSF HF membranes, thereby strengthening the repulsive interactions between the PG and foulants that carry a negative charge.^[63]^

**Table 4:** Summarized fouling parameters for raw and GO modified PSF HF membranes (the error referred to standard deviation of 3 samples).

Parameters (%)	P	PG
BSA		
R_t_	38±4	41±4
R_r_	6±1	0
R_ir_	32±3	41±4
FRR	68±7	59±6
HA		
R_t_	54±5	41±4
R_r_	37±4	33±3
R_ir_	16±2	8±1
FRR	84±8	92±9
Oil‐in‐water		
R_t_	82±8	69±7
R_r_	32±3	55±6
R_ir_	51±5	14±1
FRR	50±5	86±9
*E. coli*		
PF_1_	35±4	25±5
PF_WB_	50±5	50±5
PF_EW_	35±4	30±3
PF_2_	50±5	55±6

First of all, PG membranes did not display anti fouling performances improvement against BSA compared to P. According to Table 4, fouling parameters are quite similar from the raw PSF HF membranes with FRR and R_t_ and R_ir_ values respectively around 60 and 40 %. BSA is a complex protein which behavior is widely influenced by its environment. Several studies focused on it and can provide some information to understand the adsorption mechanism of the protein onto the substrate.[^64^, ^65^, ^66^] BSA can be adsorbed either by electrostatic or hydrophobic interactions, which means that the WCA and zeta potentials of the membrane are the key parameters. With an isoelectric point of 4.7, BSA demonstrates a negative surface charge at the experimental pH of 7.4.^[67]^ We can assume that the negative surface charge possibly was not enough to repulse the BSA and prevent it from entering the polymer matrix. Secondly, despite a significant reduction of the WCA, hydrophobic interactions may still occur as the WCA remains again more than 90° and then surface roughness is favorable to the molecules adsorption. Primarily, the significant pore size of approximately 260 nm facilitated the movement of 10 nm BSA molecules through the membrane, ultimately contributing to pore blockage. This counteracted the positive enhancements introduced to the membrane structure. This assumption is supported also by the slight and constant decrease of the flux of BSA solution through the PG membrane as depicted on Figure 4a. This phenomenon appears to be the main factor enhancing the fouling propensity towards BSA of the as‐prepared membrane. Membranes prepared with GO and lower pore size would probably give better results, but this meant completely modifying the membrane production process.

Secondly, when considering HA filtration, a notable 10 % increase in flux recovery ratio (FRR) was noted for PG in comparison to P. This increase was accompanied by a reduction in the total fouling ratio (R_t_) from 54 % to 41 %, with a particularly intriguing 50 % decrease in irreversible fouling (R_ir_). HA molecules, as indicated by DLS measurements in Table 1, are significantly larger than BSA molecules–40 times larger, to be precise. Consequently, this effectively eliminates the steric exclusion challenge encountered with BSA, stemming from the considerably disparate pore sizes of the two membranes. HA, a hydrophobic compound, carries a negative charge at pH 7.4 with a zeta potential of −40 mV.^[68]^ Initially, this might suggest that its fouling behavior should be akin to that of BSA. However, a closer examination reveals that the fouling mechanisms of BSA and HA^[69]^ on PSF membranes significantly diverge, a topic that Rabbani *et al*. delved into. According to their explanation, HA does not readily adhere to PSF membranes under a range of exposure conditions, in contrast to the ready adsorption observed for the protein BSA, primarily driven by electrostatic interactions.^[59]^ The study further elucidates that fouling caused by HA on PSF is characterized by pore blockage, whereas the adsorption of BSA onto the PSF membrane leads to irreversible fouling as a subsequent consequence.^[70]^ Hence, the improved resistance of GO‐modified PSF HF membranes to HA fouling compared to BSA can be rationalized by the greater negativity of HA molecules, prompting more robust repulsive interactions with the membranes as opposed to BSA. Moreover, the distinction in fouling mechanisms adds to this variation: HA obstructs the surface pores but can be dislodged through water backwashing, in contrast to BSA, which infiltrates the membrane matrix.

Then, PG recovered almost 90 % of water flux after oil‐in‐water emulsion filtration against 50 % for P with a 16 % reduced R_t_ lowering the irreversible fouling R_ir_ from 50 to almost 10 %. This behavior could be easily explained based on both steric exclusion, according to the droplets size registered in Table 1, and enhanced hydrophilicity. In fact, as the hydrophilicity is enhanced from 100 to 92°, the hydrophobic interactions between oil droplets are reduced and the water phase passes easier through the GO modified PSF HF membranes. On the untreated P membranes, an oil layer formed on the surface, contributing to an increase in overall resistance.^[71]^ This effect was mitigated in PG membranes due to the presence of hydrophilic groups from GO. In fact, Chackrabarty *et al*. explored the cross‐flow ultrafiltration of stable oil‐in‐water emulsions using PSF membranes. They noted that the oil rejected by the membrane amassed near the membrane surface, leading to a decline in flux driven by the concentration gradient. Furthermore, they observed that during ultrafiltration, larger oil droplets tended to settle on the membrane surface, impeding permeate flow, while smaller droplets attempted to traverse the membrane pores, causing pore blockage.^[72]^ The incorporation of GO appeared to weaken these hydrophobic interactions between the oil droplets and the membrane surface.^[73]^ This phenomenon elucidates the observed higher flux recovery ratio (FRR) and lower total fouling ratio (Rt) and irreversible fouling (Rir) in GO‐modified PSF HF membranes. These findings are particularly impressive, considering the minimal quantity of GO introduced into the membrane and the mere 10 % reduction in water contact angle (WCA).

Finally, behavior of PG and P were quite similar against *E. coli* bacteria. For both membranes, PF_1_ and PF_EW_ value were around 30 % while PF_WB_ and PF_2_ were around 50 %. Their retention performances could be explained by a simple steric phenomenon and bacteria adsorption. At the end, GO‐modified membranes (PG) exhibited high retention performances and enhanced flux properties compared to P membranes. Together with the increased pore size, a higher hydrophilicity (Table 2) of the PG surface could allow faster filtration. Nonetheless, the substantial surface roughness observed in PG membranes, as depicted in Figure 2, could foster notable affinity and adsorption of bacteria. Bernardes *et al*. concentrated on enhancing PSF membranes for water filtration by mitigating biofouling. Their investigation suggested that the modified membranes’ hydrophilicity and negative zeta potential might have contributed to the low levels of adhered cells found on these surfaces.^[74]^ In the case of GO/PES MMMs, Hu *et al*. achieved impressive fouling recovery (94.2 %) and antibacterial efficacy (90.0 %) against E. coli.^[75]^ They attributed this success to improved hydrophilicity, heightened negative charge, and the nano‐size effect of GO. Similarly, Zhang et al. elucidated that their GFG/PSF UF MMMs demonstrated exceptional antimicrobial properties and sustained effectiveness against E. coli. This was attributed to the distinctive properties of GO nanosheets, including sharp edges that fostered stronger interactions with bacterial cell membranes, leading to bacterial destruction. Additionally, they proposed that the grafting of guanidyl groups on GO nanosheets facilitated binding with phosphate groups on the cell wall through bidendate interactions, ultimately resulting in bacterial demise.^[6]^ However, in this study, no significant antimicrobial activity was observed for the GO‐modified membranes. Alternatively, if such activity had been present, the membranes might have regained some level of flux recovery after the filtration and backwashing steps.

## 4 Conclusions

This investigation focused on evaluating the efficacy of PSF HF membranes modified with GO against a range of different foulants. The introduction of GO resulted in 15 times increased pore size and almost 10° enhanced hydrophilicity, leading to a remarkable fourfold increase in permeability. For the filtration of BSA and *E. coli*, the fouling tendencies of both untreated and GO‐modified PSF HF membranes exhibited similarities. Steric exclusion emerged as the predominant phenomenon in these cases, wherein the addition of GO did not yield significant improvements in preventing BSA molecule adsorption or bacterial behavior. However, both types of membranes displayed substantial 99.9 % retention of *E. coli*, with fouling that might potentially become reversible through extended ethanol washing. Intriguingly, despite a significant increase in surface roughness, GO‐modified PSF HF membranes still demonstrated superior fouling performance compared to the untreated membranes, showcasing 10 % and 1.7 times improved flux recovery ratio (FRR) and twice and 3.6 times reduced irreversible fouling (R_ir_) respectively during the filtration of humic acid and oil‐in‐water. This could be attributed to the more negatively charged surface and diminished hydrophobicity achieved with GO incorporation. Furthermore, the integration of GO into PSF HF membranes notably enhanced their ability to reject oil, rendering them well‐suited for applications in oil/water separation. Exploring the potential of these membranes for filtering and separating other contaminants or diverse emulsions would be an interesting avenue for further study. Furthermore, investigating the fouling mechanisms that arise during the filtration of various contaminants would significantly enhance this study. This could be achieved by observing changes in the zeta potential of the membrane during filtration, which would offer new insights into fouling behavior and understanding.

## Conflict of Interests

The authors declare no conflict of interest.

5

## Supporting information

As a service to our authors and readers, this journal provides supporting information supplied by the authors. Such materials are peer reviewed and may be re‐organized for online delivery, but are not copy‐edited or typeset. Technical support issues arising from supporting information (other than missing files) should be addressed to the authors.

Supporting Information

## Data Availability

The data that support the findings of this study are available from the corresponding author upon reasonable request.
