# Supplementary material for: Membranes Fouling Propensity of PSF/GO Hollow Fiber Mixed Matrix Membranes for Water Treatment Ultrafiltration Application
Source: ChemSusChem. 2024 Nov 28;18(7):e202401061. doi: 10.1002/cssc.202401061 (PMC11960592; doi:10.1002/cssc.202401061)
Supplement: Supplementary file 1 — Supporting Information [file CSSC-18-e202401061-s001.pdf]

ChemSusChem

Supporting Information

Membranes Fouling Propensity of PSF/GO Hollow Fiber Mixed Matrix Membranes for Water Treatment Ultrafiltration Application

Jeanne Casetta, Héloïse Baldo, Laurence Soussan, Céline Pochat-Bohatier,*
Mikhael Bechelany,* and Philippe Miele

Membranes fouling propensity of PSF/GO hollow fiber mixed matrix membranes for water treatment ultrafiltration application

Jeanne Casetta [1], Héloïse Baldo[1], Laurence Soussan[1], Céline Pochat-Bohatier [1]*, Mikhael Bechelany [1,2]*, Philippe Miele [1]

[1]Institut Européen des Membranes, IEM, UMR-5635, Univ Montpellier, ENSCM, CNRS, Place Eugene Bataillon, 34095 Montpellier, France
[2] Gulf University for Science and Technology, GUST, Kuwait
* celine.pochat@umontpellier.fr and mikhael.bechelany@umontpellier.fr

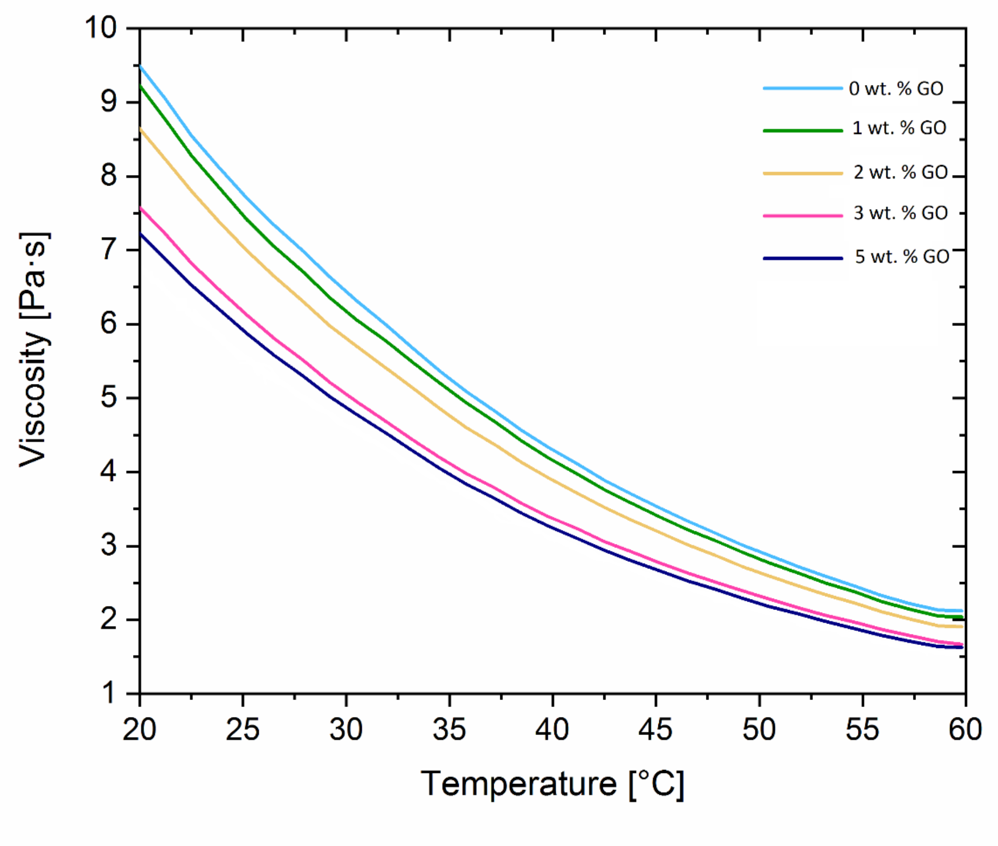

Figure S1. Viscosity over temperature of 0 to 5 wt. % GO polymer solutions performed with the rheometer MCR 301 Anton Paar device. All of the experiments were run using a cone-plate (CP50-1) geometry with a 50 mm diameter and a gap set in the center of the cone at 101µm. Shear rate range was fixed at 0.01-1000 s^{-1}. Silicon oil was placed around the geometry to avoid a possible evaporation during the experiment.

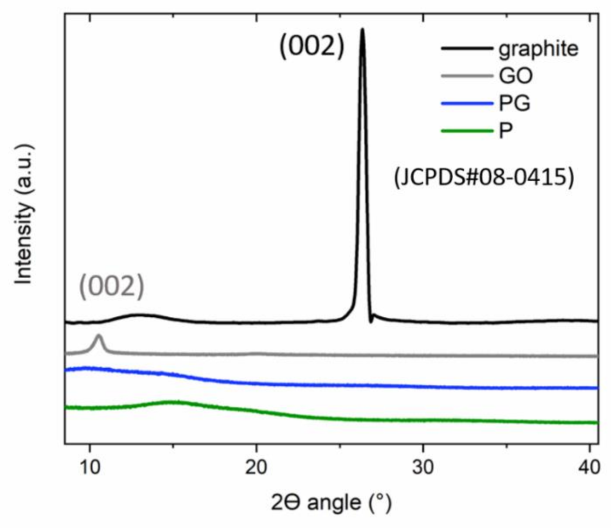

Figure S2. XRD spectra of graphite, GO, PSF HF (P) and PSF GO-modified HF (PG) membranes. The analyses are made using a round holder, PANalytical Xpert system with Cu Kα radiation (λ = 0.15406 nm), 2θ range between 5 and 45° and a step rate of 0.02°/sec.

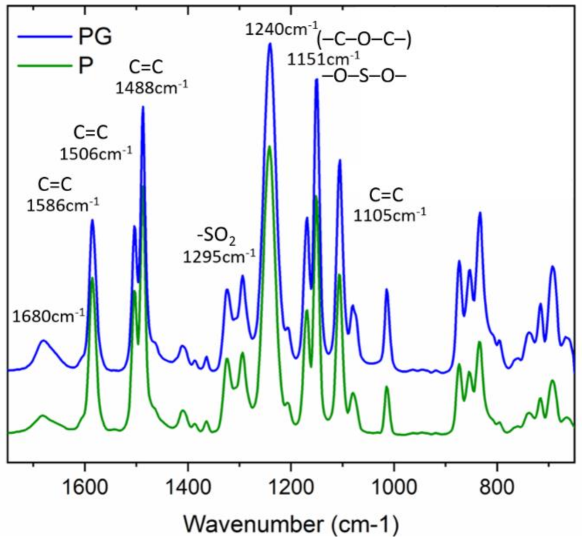

Figure S3. ATR-FTIR spectra of raw P and GO modified PG PSF HF membranes. The analyses are made on membranes previously washed and dried with ethanol, n-hexane and a 50°C oven overnight. The ATR-FTIR spectra were recorded across the frequency range of 600-4000cm^{-1} with a resolution of 4 cm^{-1} and data were averaged from 64 scans.
